# Mitochondrial Kinase Signaling for Cardioprotection

**DOI:** 10.3390/ijms25084491

**Published:** 2024-04-19

**Authors:** Kerstin Boengler, Chantal Eickelmann, Petra Kleinbongard

**Affiliations:** 1 Institute of Physiology, Justus-Liebig University, 35392 Giessen, Germany; 2 Institute for Pathophysiology, West German Heart and Vascular Center, University of Essen Medical School, 45147 Essen, Germany; chantal.eickelmann@uk-essen.de (C.E.); petra.kleinbongard@uk-essen.de (P.K.)

**Keywords:** mitochondria, protein kinase, cardioprotection, preconditioning, ischemia, reperfusion

## Abstract

Myocardial ischemia/reperfusion injury is reduced by cardioprotective adaptations such as local or remote ischemic conditioning. The cardioprotective stimuli activate signaling cascades, which converge on mitochondria and maintain the function of the organelles, which is critical for cell survival. The signaling cascades include not only extracellular molecules that activate sarcolemmal receptor-dependent or -independent protein kinases that signal at the plasma membrane or in the cytosol, but also involve kinases, which are located to or within mitochondria, phosphorylate mitochondrial target proteins, and thereby modify, e.g., respiration, the generation of reactive oxygen species, calcium handling, mitochondrial dynamics, mitophagy, or apoptosis. In the present review, we give a personal and opinionated overview of selected protein kinases, localized to/within myocardial mitochondria, and summarize the available data on their role in myocardial ischemia/reperfusion injury and protection from it. We highlight the regulation of mitochondrial function by these mitochondrial protein kinases.

## 1. Introduction

Mitochondria have important roles in the maintenance of normal cell function; therefore, a dysregulation of mitochondrial functions consequently accounts for a variety of human disease pathologies including infectious and inflammatory diseases, pulmonary diseases, neurodegenerative diseases, and cardiovascular diseases (for a review, see [1]). In the present review, we focus on a personal and opinionated overview of the mitochondrially localized protein kinases potentially involved in mitochondrial signaling in myocardial damage by ischemia and reperfusion (I/R) and the strategies to minimize such myocardial injury.

In I/R injury, the death of cardiomyocytes and non-cardiomyocytes is based on necrosis, apoptosis, necroptosis, pyroptosis, and autophagy. The contribution of the different modes of cell death to infarct size is unclear at present (for a review, see [2]). To prevent myocardial cell death due to ischemia, it is essential to reperfuse the myocardium in a timely manner. However, the restoration of blood flow itself causes additional damage to the myocardium [3]. The high number of patients who suffer myocardial infarction makes it necessary to develop strategies to reduce myocardial I/R damage [4]. Among these strategies is ischemic conditioning, which is defined as the infarct size reduction by nonlethal periods of I/R, which are executed at different points in time in relation to the sustained phase of I/R. In ischemic preconditioning (IPC), the nonlethal periods of I/R are carried out before, in ischemic perconditioning during, and in ischemic postconditioning (IPostC) after the sustained phase of ischemia, which is followed by reperfusion. In contrast to the aforementioned cardioprotective maneuvers, in remote ischemic conditioning (RIC), the short periods of ischemia are not applied directly to the heart, but to other tissues such as skeletal muscle [5]. In classical conditioning, the nonlethal I/R cycles are performed in a close temporal context (minutes) to the index ischemia, whereas in late conditioning, 24–48 h are left between the short cycles of I/R and the index ischemia. In contrast to classic preconditioning, late preconditioning involves the de novo synthesis of proteins, e.g., of the manganese superoxide dismutase (MnSOD) [6,7] or protein kinase C (PKC) [8]. Besides these mechanical approaches, pharmacological interventions are also known to reduce myocardial infarct size [2,9]. However, the strongest and most robust cardioprotective intervention, which is effective in all species tested so far, including humans, is mechanically induced ischemic conditioning [2,10].

The cardioprotective maneuvers activate signaling cascades (see below) and finally target the mitochondria, where they modify the function of the organelles [11,12,13,14]. The functional parameters affected by cardioprotective maneuvers include the preservation of respiration and ATP production, the reduction in reactive oxygen species (ROS) release at the onset of reperfusion, the inhibition of mitochondrial permeability transition pore (MPTP) opening at the beginning of reperfusion, the preservation of mitochondrial morphology, and the activation of mitophagy. For details on mitochondrial function in I/R injury, the reader is referred to recent articles [15,16,17,18,19,20]. Taken together, the cardioprotective maneuvers of IPC, RIC, or IPostC target the mitochondria and alter the function of the organelles. As a result, mitochondrial function is preserved, which finally enhances cell viability after I/R injury.

## 2. Cardioprotective Signaling to and within Mitochondria

Niemi and Pagliarini show that 91% of annotated mitochondrial proteins within MitoCarta3.0 have at least one phosphorylation site [21]; however, only about 5% of these sites are associated with published studies. Proteomic studies identified 77 phosphoproteins within different mitochondrial compartments of human skeletal muscle [22], 184 phosphoproteins in rodent myocardial mitochondria [23], 181 phosphoproteins in murine mitochondria [24], and a total of 354 phosphoproteins in mitochondria isolated from rat liver, heart, and muscle [25]. Whereas protein phosphorylation is a prerequisite for the import into mitochondria [21], it is also suggested that the phosphorylation may take place within the organelles. This hypothesis is supported by the identification of protein kinases within isolated mitochondria. For example, 52 protein kinases are detected in mitochondria isolated from the rat insulinoma cell line INS-1 [26], whereas 25 protein kinases are detected within mitochondria isolated from rat liver, heart, and muscle [25]. Studies not only detect kinases in the organelles, but also protein phosphatases, which regulate the activity of their target proteins. In fact, 12 protein phosphatases with distinct catalytic domains to confer substrate selectivity are known to be located within mitochondria of murine origin [21].

**Figure 1:**
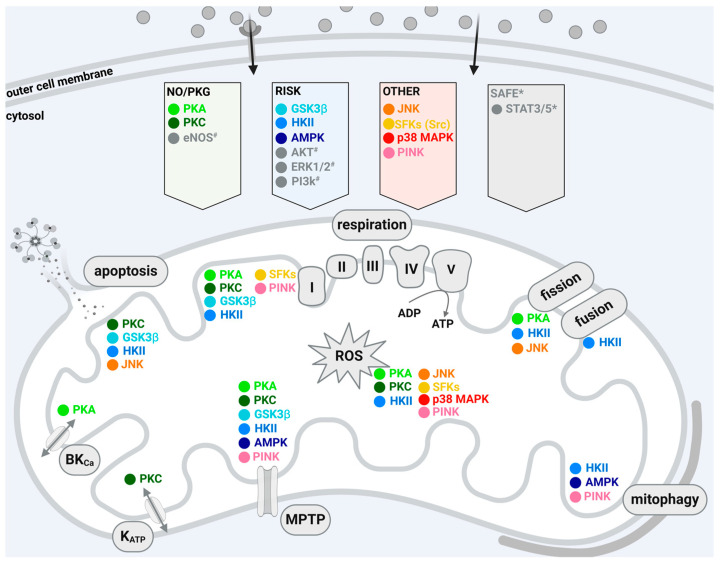
Schematic overview of signaling pathways activated by cardioprotective adaptations and of mitochondrially associated and/or localized kinases and their effects on mitochondrial function. The figure summarizes the main cardioprotective signaling pathways. Released or generated extracellular molecules (gray dots) act on sarcolemmal receptors or act receptor-independently, and subsequent downstream cytosolic signaling cascades are activated: the NO/PKG, RISK, and SAFE pathways, as well as other kinases not associated with the indicated pathways. Pathways/kinases displayed in gray are not included in the present review. The kinases within the mitochondrion in the lower part of the figure are shown according to their influence on mitochondrial function in cardioprotective interventions, not according to their localization within the organelles. Created with BioRender.com. Abbreviations: I, II, II, IV, V (ATP synthase) indicate respiratory chain complexes; ADP: adenosine diphosphate; AKT: protein kinase B; AMPK: adenosine monophosphate-activated protein kinase; ATP: adenosine triphosphate; BK_Ca_: Ca^2+^-activated potassium channel; ERK: extracellular signal-regulated kinase; GSK3β: glycogen synthase kinase 3β; HK II: hexokinase II; JNK: C-Jun N-terminal kinase; K_ATP_: ATP-dependent potassium channel; MPTP: mitochondrial permeability transition pore; NO: nitric oxide; PI3k: phosphoinositide 3-kinase; p38 MAPK: p38 mitogen-activated protein kinase; PINK: PTEN-induced putative kinase 1; PKA: protein kinase A; PKC: protein kinase C; PKG: protein kinase G; RISK: reperfusion injury salvage kinase pathway; ROS: reactive oxygen species; SAFE: survivor activating factor enhancement pathway; SFK: Src-family protein tyrosine kinases; STAT3/5: signal transducer and activator of transcription 3/5; # protein kinases involved in the cardioprotective signaling pathways, but not selected based on the mentioned criteria (see text); * relevant cardioprotective signaling pathway, but not further mentioned as its members do not meet the criteria indicated in the text.

Cardioprotective maneuvers mediate the reduction of I/R injury through the release or generation of extracellular molecules that act on sarcolemmal receptors or act receptor-independently and the subsequent activation of downstream cytosolic signaling cascades [4,13]. The different signal transduction pathways can be grouped in three main cascades, which are termed the RISK pathway (reperfusion injury salvage kinase pathway), including protein kinase B (AKT) and extracellular signal-regulated kinase (ERK), glycogen synthase kinase 3β (GSK3 β) [27,28,29,30], SAFE pathway (survivor activating factor enhancement pathway), including tumor necrosis factor α and signal transducer and activator of transcription 3 [31,32,33], and a pathway including protein kinase A (PKA), nitric oxide (NO), protein kinase G (PKG), and PKC [34]. A schematic overview of the kinases involved in the indicated pathways is presented in Figure 1. It is a common feature of these signaling pathways, which can also interact with each other, that they converge on the mitochondria and modify their function [12,13]. In addition, there are other protein kinases not directly attributed to these classical protective pathways, which also confer cardioprotection by targeting mitochondria such as Src-family protein tyrosine kinases (SFKs), hexokinase, adenosine monophosphate-activated protein kinase (AMPK), C-Jun N-terminal kinase (JNK), p38 mitogen-activated protein kinase (p38 MAPK), and PTEN-induced putative kinase 1 (PINK1).

In our review, we highlight the role of protein kinases, which are located within mitochondria and contribute to myocardial I/R injury or protection from it via the modulation of mitochondrial function. We focus on protein kinases, selected based on the following criteria: (1) a localization of the protein kinase within cardiac mitochondria is demonstrated; (2) the protein kinase is known to be involved in signaling pathways contributing to I/R injury and the protection from it; (3) data indicate an influence of the mitochondrial fraction of the kinase on mitochondrial functional parameters important for I/R injury. The order in which the kinases are discussed is based on their affiliation to the signal transduction pathways stated above (i.e., the RISK and NO/PKG pathway; please note that the SAFE pathway is not further mentioned, as its members do not meet the criteria indicated above) or on their interaction with a kinase attributed to such pathway (even if it is unclear, if the mitochondrial fraction of the protein is part of the same pathway). If a kinase cannot be assigned to such a classical signaling pathway, we describe this accordingly. As the contribution of the mitochondrial protein kinases discussed in the present review towards cell death have not been systematically analyzed, we cannot present data on all forms of cell death contributing to I/R injury.

We provide an overview of the localization, translocation, and protein–protein interactions of these mitochondrial protein kinases (Table 1) and summarize their effects on mitochondrial function in the context of I/R injury in the following chapters. Again, this is not a comprehensive review of kinases targeting mitochondrial function; this is an opinionated review focusing on those publications characterizing a cardioprotective kinase function in mitochondria.

## 3. Protein Kinases of the NO/PKG Pathway

The following section of the manuscript deals with the function of mitochondrial PKA and PKC as kinases assigned to the protective NO/PKG pathway.

### 3.1. Protein Kinase A (PKA)

PKA or cAMP-dependent protein kinase is causally involved in cardioprotection by IPC [67,68,69] and nitrite [39] in rodent hearts. Studies on a PKA participation in RIC or IPostC are not yet available. PKA activation is triggered through G protein-coupled receptors, and PKA is upstream of PKC in the cardioprotective cytosolic nitric oxide/PKG pathway [2,13,70]. In rodent hearts, PKA activates the endothelial nitric oxide synthase (eNOS) [69], and the PKA activation increases the phosphorylation of the cAMP response element-binding protein [68]. Also, in humans and mice, I/R injury induces oxidation and disulfide formation of the regulatory subunit Iα-containing protein kinase A, which finally reduces lysosomal two-pore channel-dependent calcium release and thereby limits infarct size [71].

In rodent myocardium, PKA is recruited via its regulatory subunits and special anchoring proteins—the PKA-anchoring proteins (AKAP)—to the mitochondrial outer membrane [35,36,72]. PKA subunits are also detected within the inner mitochondrial membrane and the matrix fraction of subfractionated mitochondria from rat heart [38], bovine heart, and mouse myoblasts [37]. An enrichment of PKA is demonstrated in subsarcolemmal mitochondria isolated from mouse ventricular tissue [73]. PKA stimulation affects mitochondrial function through the glycogen synthase kinase 3β (GSK3β)-dependent inhibition of MPTP opening [74,75]. Also, PKA attenuates the mitochondrial Ca^2+^ overload via the cAMP-dependent protein-kinase-mediated opening of mitochondrial calcium-sensitive potassium channels [76,77]. In isolated mitochondria, PKA activates proteins of the respiratory chain complex I [37]; in permeabilized cardiomyocytes, PKA regulates the mitochondrial redox state and the mitochondrial membrane potential via mitochondrial ROS generation [78]. Activated PKA phosphorylates mitochondrial Drp1 at serine 637 [39] in rodent hearts and Drp1 phosphorylation prevents I/R-induced mitochondrial fission [79]. A deficiency of the PKA-anchoring protein AKAP1 promotes mitochondrial aberrations and exacerbates cardiac injury following permanent coronary ligation via enhanced mitophagy and apoptosis [80]. In human myocardium, PKA is also described to be co-localized with mitochondria [71]; however, whether this interaction is also relevant for cardioprotection is unknown [81].

A fraction of PKA, which contributes to cardioprotection by IPC, resides within cardiac mitochondria, although data on its role in cardioprotection by RIC or IPostC are not yet available. However, data on the submitochondrial localization of the protein are not consistent and more detailed studies are needed to precisely characterize the submitochondrial PKA localization. The available studies show that PKA affects several parameters of mitochondrial function including respiration, ROS formation, MPTP opening, fission, and mitophagy and may, therefore, contribute to cardioprotection, but a precise role of the mitochondrially localized protein in the context of cardioprotection has not been demonstrated yet.

### 3.2. Protein Kinase C (PKC)

In response to ischemic conditioning, PKC is activated and PKC activation is causally involved in cardioprotection by IPC [82,83] and late IPC [8], IPostC [84,85] and nociceptive remote conditioning [86], whereas PKC is unlikely to be involved in the protective effects of RIC [87]. In porcine myocardium, the inhibition of PKC with staurosporine does not result in a loss of cardioprotection by IPC [88], whereas the combination of staurosporine and genistein to inhibit protein tyrosine kinase abolishes the infarct size reduction by IPC [89]. PKC is an element of the cardioprotective cytosolic NO/PKG pathway, which interacts with the RISK pathway. Thus, several alternatives for activating PKC are known [2,13,70]. In response to the activation of G-protein-coupled receptors, PKC is directly activated (i.e., via adenosine) or phosphatidylinositol 3-kinase/AKT is activated, which then results in the activation of eNOS, NO production, guanylate cyclase activation, and PKG and PKC activation. The three isoforms of the PKC (PKCα, PKCε, and PKCδ), however, appear to be involved in a species-dependent manner. The activation of PKCα in response to IPC-mediated protection is responsible in dogs and pigs through the activation of ecto 5′ nucleotidase and adenosine formation [90] and interaction/colocalization with sarcolemmal Connexin 43 (Cx43) [91]. The PKCε isoform is involved in cardioprotection by IPC [92,93] and IPostC [84,85] in rodents. Controversial results exist regarding the PKCδ isoform. In rodent hearts, PKCδ knockout abolishes IPC’s cardioprotection [94], whereas in pigs, the selective pharmacological blockade of PKCδ is associated with cardioprotection [95].

The activated PKCε isoform translocates through a heat-shock-protein-translocase of the outer membrane (TOM) 20-interaction to mitochondria [40], and TOM70 is also implicated in this process [42]. PKCε resides in mitochondria isolated from neonatal rat cardiomyocytes [44,96] and mouse [45] and rabbit [97] myocardium. The analysis of subfractionated mitochondria indicates PKCε at the inner mitochondrial membrane [40] or in a protein pool consisting of inner mitochondrial membrane and matrix proteins [41]. Mitochondrial PKCε interacts with JNK, p38 MAPK, and ERK. The formation of PKCε/ERK complexes inactivates the pro-apoptotic protein Bad (B cell lymphoma (BCL)2-associated agonist of cell death) and thereby exerts cardioprotective effects [43]. PKC activation results in the opening of the mitochondrial ATP-dependent K channel (mitoK_ATP_). The influx of potassium ions via the mitoK_ATP_ triggers modest ROS formation by respiratory chain complex I, which then, in turn, results in p38 MAPK and PKC activation [97,98] and, finally, prevents MPTP opening [99]. The ROS formation induced by mitoK_ATP_ opening with diazoxide and the protection afforded by diazoxide are dependent on the presence of Cx43 [100]. The cardioprotection by IPC is also dependent on the presence of Cx43 [101]. A small fraction of the gap junction protein Cx43 resides within cardiac subsarcolemmal mitochondria [102,103] and influences mitochondrial function in terms of respiration, ROS formation, and MPTP opening [102]. Mitochondrial Cx43 is phosphorylated at several residues, whereby the phosphorylation by casein kinase 1 is central for the cardioprotection by IPC [104]. However, it is unclear at present whether Cx43 is a target of mitochondrially localized protein kinases or if the phosphorylation of the protein is a prerequisite for its mitochondrial import.

The addition of recombinant PKCε to isolated cardiac mitochondria inhibits MPTP opening, and this effect may be mediated by the interaction of PKCε with proteins modulating the MPTP such as VDAC1, ANT, and HKII [45]. Complex IV of the electron transport chain (cytochrome c reductase) is positively regulated by the cAMP-dependent action of the PKCε and PKCε co-immunoprecipitates with the cytochrome c oxidase subunit IV [44]. The protein–protein interaction seems to be relevant for improved energetics following hypoxic preconditioning. Also, IPC [97,105]), isoflurane-induced preconditioning [106], and adenosine treatment [42] increase the mitochondrial amounts of PKCε.

Not only PKCε but also PKCδ resides in rodent mitochondria [105,107]. The mitochondrial translocation of PKCδ occurs during the reperfusion, and the inhibition of PKCδ decreases ROS formation and enhances mitochondrial respiration after I/R [108]. The increase in mitochondrial PKCδ upon reperfusion stimulates the release of cytochrome c and propagates apoptosis [107]. In line with the aforementioned data are studies demonstrating that simultaneous PKCε activation and PKCδ inhibition seem to amplify the effect of myocardial protection [109] and that the mitochondrial PKC isoform ratio is regulated by cellular ROS levels [107]. The increase in mitochondrial PKCε at the end of reperfusion in preconditioned rat hearts is paralleled with a reduction in mitochondrial PKCδ [110].

In isolated human right atrial trabeculae, PKC-activated protection is abrogated by a K_ATP_ antagonist [111], and PKCε activation is supposed to be upstream and PKCα downstream of the mitoK_ATP_ channels [112]. The translation of PKC-dependent cardioprotection to patients, however, has not been successful so far. Initial results on pharmacological PKCδ inhibition in the preclinical situation seem promising. In rodent hearts, KAI-9803 inhibits PKCδ activity and prevents the translocation of this PKC isoform to the mitochondria, which preserves mitochondrial function [107]. In a pig model, the intracoronary administration of KAI-9803 prior to reperfusion reduces infarct size [95], and the PKCδ inhibitor deltaV1-1 attenuates microvascular dysfunction at reperfusion [113]. In a clinical trial, however, the inhibition of PKCδ by delcasertib as an adjunct to reperfusion therapy during the primary percutaneous coronary intervention of patients with ST-elevation myocardial infarction (PROTECTION AMI Randomized Controlled Trial) failed to induce cardioprotection. There was no change in creatine kinase–muscle band release [114].

**Figure 2:**
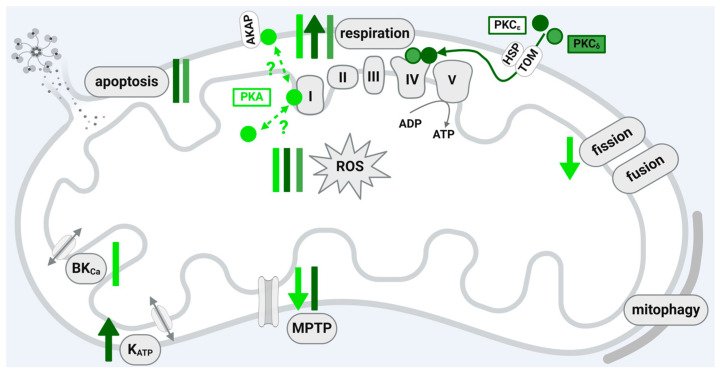
Effects of mitochondrially localized protein kinases associated with the NO/PKG pathway on mitochondrial function of cardiomyocytes or cardiomyocyte cell lines. The figure summarizes the effects of mitochondrially localized protein kinase A (PKA; light green) and the protein kinase C isoforms ε (PKCε; dark green) and δ (PKCδ; grass green) on mitochondrial function of cardiomyocytes or cardiomyocyte cell lines. Arrows pointing upwards indicate an activating effect upon a cardioprotective stimulus; arrows pointing downwards indicate an inhibiting effect upon a cardioprotective stimulus; lines without an arrowhead indicate influence on mitochondrial function without cardioprotective stimulus; light green dotted arrows with question marks refer to alternative localizations of PKA at the inner membrane or matrix, respectively. For further details, see Table 2. Created with BioRender.com. Abbreviations: I, II, II, IV, V (ATP synthase) indicate respiratory chain complexes; ADP: adenosine diphosphate; AKAP: PKA-anchoring protein; ATP: adenosine triphosphate; BK_Ca_: Ca^2+^-activated potassium channel; HSP: heat shock protein; K_ATP_: ATP-dependent potassium channel; MPTP: mitochondrial permeability transition pore; ROS: reactive oxygen species; TOM: translocase of the outer membrane.

In sum, depending on the subfractionation method, PKC is detected in rodent hearts in the inner mitochondrial membrane and in the mitochondrial matrix. Here, the translocation of PKCε to the mitochondria affects the function of the organelles and thereby contributes to cardioprotection (see Figure 2). The interaction of PKCε with other kinases also localized within mitochondria is involved in the prevention of I/R injury. In addition to PKCε, PKCδ is also detected in rodent mitochondria, where it exerts opposing effects to those of PKCε. Therefore, the simultaneous activation of PKCε and inhibition of PKCδ is hypothesized to be more effective in minimizing myocardial I/R damage than the activation/inhibition of one isoform alone. The role of mitochondrial PKC in species other than rodents is not clear, and the translation to human myocardium has failed so far.

A scheme that summarizes the influences of PKA and PKC isoforms localized within the mitochondria of cardiomyocytes or cardiomyocyte cell lines on the function of the organelles is shown in Figure 2.

## 4. Protein Kinases of the RISK Pathway

In the subsequent part of the manuscript, we describe the role of mitochondrial GSK3β as a protein of the RISK pathway. Furthermore, the function of mitochondrial kinases such as hexokinase II (HKII) and adenosine monophosphate-activated protein kinase (AMPK) in the context of myocardial I/R injury is described, as these kinases influence the activity of GSK3β, although they are not part of the classical RISK pathway. AKT, which is part of the RISK pathway and described to be located in mitochondria, is discussed in the context of its interaction with HKII.

### 4.1. Glycogen Synthase Kinase 3β (GSK3β)

The serine/threonine kinase GSK3 is ubiquitously expressed and highly conserved in eukaryotes and has been shown to regulate glycogen metabolism. The finding that GSK3 is a kinase with a high number of substrates [115] implies that its function is more diverse than originally described. In the heart, GSK3 is involved, for example, in the development of fibrosis [116], hypertrophy [117], and heart failure [118,119]. GSK3 is expressed in two isoforms, i.e., GSK3α and GSK3β, which show an overall homology of about 85%. Whereas GSK3α and GSK3β share some functions, the isoforms also demonstrate some unique properties [120,121,122]. GSK3 differs from several other kinases in the way that it is mostly active under unstimulated conditions, but becomes inactivated by phosphorylation upon different forms of input including myocardial I/R injury. The major phosphorylation site of GSK3β is serine 9 for the negative regulation, whereas tyrosine phosphorylation at tyrosine 216 positively regulates GSK3β activity [123]. GSK3β is part of several signal transduction pathways and is involved in the signaling induced by kinases such as AKT, which phosphorylates GSK3β at serine 9, or AMPK, i.e., kinases, for which a mitochondrial localization has been described (see text and [50]) and that contribute to myocardial I/R injury and the protection from it. I/R injury induces a dephosphorylation of GSK3β at serine 9 and thereby activates the kinase in rat hearts in vivo [124] and in vitro [125]. Accordingly, the inhibition of GSK3β by SB216763 decreases myocardial I/R injury in isolated rat [126,127] and mouse hearts [128,129]. A recent study shows that the inhibition of GSK3β by direct binding of neopetroside A protects the heart against myocardial I/R damage [130]. However, the effects of GSK3β appear to be dependent on whether they are characterized after myocardial ischemia alone or after I/R [131]. Phosphorylation and, thereby, the inhibition of GSK3β is induced by IPC in mouse [132] and rat hearts [129] in vitro, by IPostC in rats hearts in vitro [125] and in vivo [133], and by RIC in isolated rat hearts [134]. However, one study shows that IPC and IPostC in mouse hearts in vitro fail to induce GSK3β phosphorylation after ischemia or I/R [135]. Regarding the cardioprotection by pharmacological preconditioning, GSK3β is increasingly phosphorylated at serine 9 upon the administration of rosuvastatin, sevoflurane, or triiodothyronine [124,136,137]. While IPostC fails to decrease myocardial infarction in mice in which serine 9 within GSK3β is mutated to alanine (GSK3β-S9A mice) [128], IPC and IPostC effectively reduce infarct size in mice in which serine 9 of GSK3β and serine 21 of GSK3α are rendered to non-phosphorylatable residues [135]. The discrepancies between these data are discussed in an article by Murphy and Steenbergen [138]. The authors hypothesize that the already reduced infarct size in the mouse line with a knockin of GSK3α and GSK3β that cannot be phosphorylated (studied by Nishino et al. [135]) indicates an activation of protective signaling pathways. If such signaling pathways act downstream of GSK, they can be effectively induced by IPC or IPostC and may confer cardioprotection even in the presence of non-phosphorylatable GSK3β.

Whereas GSK3β is present in the cytosol, a certain fraction of the protein localizes to the mitochondria. Mitochondrial translocation of the protein is induced by ROS and requires GSK3β kinase activity. Also, the interaction between GSK3β and VDAC2, which can be phosphorylated by GSK3β [46], is involved in the mitochondrial import of GSK3β [47]. In the myocardium, GSK3β resides in similar amounts in subsarcolemmal and interfibrillar mitochondria, two mitochondrial subpopulations that differ in respiration and MPTP opening [139,140,141]. In relation to myocardial I/R injury, the mitochondrial amounts of total and serine-9-phosphorylated GSK3β increase after reperfusion compared to the pre-ischemic values [48]. The phosphorylation of mitochondrial GSK3β is regulated by PKCδ, since PKCδ inhibition enhances the amounts of serine-9-phosphorylated GSK3β within cardiac mitochondria at 30 min of reperfusion [142]. The prolongation of reperfusion to 60 min is sufficient to stimulate the phosphorylation of mitochondrial GSK3β in the absence of the PKCδ inhibitor. Pharmacological preconditioning with diazoxide, which provides cardioprotection by opening mitoK_ATP_ channels, enhances the mitochondrial amounts of serine-9-phosphorylated GSK3β within cardiomyocytes under control conditions [74] and after I/R [127]. These data are confirmed using nicorandil as a mitoK_ATP_ channel opener [143]. The effects of serine-9-phosphorylated and thereby inactivated GSK3β on mitochondrial function include the inhibition of apoptosis, the enhancement of mitochondrial biogenesis and mitochondrial dynamics, and, especially, the inhibition of MPTP opening [74,144,145]. In this respect, the protein–protein interaction between GSK3β and the ANT, which is suggested to be a component of the MPTP [145,146], may be important [48]. Mitochondria isolated from GSK3β-S9A mice or wildtype mice with pharmacological GSK3β inhibition display no delayed MPTP opening after IPostC [128]. The inhibition of MPTP opening by GSK3β involves another kinase located within mitochondria, hexokinase II (HKII), which we focus on in the next section of this review. In addition, GSK3β is involved in mitochondrial bioenergetics (reviewed in [144]). The protein decreases the activity of the complexes of the electron transport chain, leading to diminished ATP and increased ROS formation. Accordingly, mitochondria from GSK3β-S9A mice demonstrate increased ADP-stimulated respiration [128]. In line with this are data showing that the GSK3β inhibitor neopetroside A increases ATP-linked respiration and concomitantly elevates cellular ATP levels [130]. Whereas the cytoprotective role of GSK3β in the protection from I/R injury is mainly attributed to the inhibition of MPTP opening, the GSK3β inhibitors MLS2776 and MLS2779 minimize myocardial I/R injury independently from targeting MPTP opening and mitochondrial GSK3β amounts in isolated murine hearts [139]. These data suggest that GSK3β may exert cardioprotective functions independent of the inhibition of MPTP opening.

In summary, the inhibition of GSK3β activity via serine 9 phosphorylation decreases myocardial I/R injury. Such phosphorylation is induced by cardioprotective maneuvers and by pharmacological preconditioning. The translocation of GSK3β into the mitochondria affects the function of the organelles in several aspects important for cell survival following I/R injury, especially the inhibition of MPTP opening at reperfusion. In the cardioprotective signal transduction cascades, GSK3β is interconnected with other protein kinases for which a mitochondrial localization is described, e.g., AMPK or AKT. Whether the protein–protein interactions between GSK3β and the other kinases take place inside or outside the mitochondria is currently unknown. The precise elucidation of the role of mitochondrial GSK3β and its interactions with other proteins is required to develop strategies to reduce I/R damage.

### 4.2. Hexokinase II (HKII)

The phosphorylation of glucose to glucose-6-phosphate by hexokinase (HK) is the first step in glucose metabolism and glucose-6-phosphate serves as a precursor for glycolysis, glycogenesis, the pentose phosphate pathway, and the hexosamine biosynthetic pathway [147]. Among the HK isoforms expressed in mammals, HKI, together with HKII, is found in the heart [148]. Here, HKI predominates in neonatal rat ventricular cardiomyocytes, whereas HKII is the major isoform in adult rat ventricular cardiomyocytes [148]. HKI and HKII differ in their subcellular localization and function: HKI is predominantly present in mitochondria and promotes glycolysis, while HKII shuttles between cytosol and mitochondria and exerts diverse functions including the supply of glucose-6-phosphate for glycogen and pentose phosphate pathways (cytosolic form) as well as for glycolysis and oxidative phosphorylation (mitochondrial form) [148,149]. The hydrophobic domain in the aminoterminus of the HKs is important for the mitochondrial localization of the proteins [150]. Due to its predominant expression in adult cardiomyocytes, in our review, we focus on the role of HKII.

Within the mitochondria, HKII interacts with VDAC present in the outer membrane, making it likely that HKII also localizes to the outer mitochondrial membrane [49,52]. The knockout of VDAC in H9C2 cells diminishes mitochondrial HKII [151]. The shuttling of HKII between the cytosol and the mitochondria is, in part, regulated by phosphorylation. HKII is targeted by AKT, which phosphorylates the protein at threonine 473 [51]. Since mitochondrial amounts of AKT increase in response to insulin-like growth factor treatment, an effect associated with enhanced mitochondrial HKII, it is possible that the interaction between AKT and HKII occurs within the organelles and increases the mitochondrial binding of HKII [51]. In line with this hypothesis are data showing that the addition of recombinant kinase-active AKT to mitochondria isolated from the mouse hearts stimulates the phosphorylation of HKII. The HKII phosphorylation, in turn, decreases the Ca^2+^-induced release of cytochrome c from the mitochondria, which is a hallmark of mitochondrial apoptosis [50]. Another kinase involved in mitochondrial HKII is GSK3β, which upon pharmacological inhibition prevents and upon activation enhances the mitochondrial dissociation of HKII [126]. Consequently, accelerating the mitochondrial HKII dissociation enhances and maintaining mitochondrial HKII attenuates MPTP opening and the loss of mitochondrial membrane potential induced by GSK3β in permeabilized myocytes. A reduction in the mitochondrial calcium retention capacity indicative of enhanced MPTP opening by the dissociation of mitochondrial HKII is also confirmed in HeLa cells and adult rat cardiomyocytes [152]. However, the knockout of cyclophilin D, which is known to facilitate MPTP opening [153], does not alter mitochondrial amounts of HKII or mitochondrial HKII activity under physiological conditions [154]. In accordance with the hypothesis that the loss of mitochondrial HKII enhances MPTP opening are data showing that the overexpression of HKII in neonatal rat cardiomyocytes protects against H_2_O_2_-induced MPTP opening.

The finding that, under physiological conditions, the reduction in mitochondrial HKII results in accelerated MPTP opening suggests that the dissociation of HKII from the organelles may be affected by myocardial I/R injury and the protection from it. Indeed, mitochondrial HKII levels decrease by simulated ischemia in neonatal rat cardiomyocytes in vitro and after ligation of the left anterior descending coronary artery in vivo [155]. In contrast, one study presents increased mitochondrial translocation of HKII induced by ischemia in isolated rat hearts [156]. The mitochondrial amounts of HKII decrease when ischemia is followed by reperfusion in vitro or in vivo [157,158]. The detailed analysis of HKII in mitochondrial subpopulations shows similar reductions of HKII in subsarcolemmal, interfibrillar, and perinuclear mitochondria at reperfusion [159]. Moreover, mitochondrial HKII decreases in coronary microvascular endothelial cells isolated after I/R in mouse hearts in vivo [160]. The importance of mitochondrial HKII levels for I/R injury is strengthened by data showing that the dissociation of mitochondrial HKII renders a non-injurious I/R stimulus into an injurious one [161]. Plotting infarct size against the end-ischemic mitochondrial HK activity results in a negative correlation between the parameters, thereby emphasizing the importance of preserved mitochondrial HKII activity for cardioprotection [162]. The mechanism by which HKII contributes to reduced myocardial I/R damage may comprise either direct or indirect MPTP inhibition. The direct inhibition of MPTP opening by HKII may involve interference with the mitochondrial binding of the proapoptotic protein Bax [163], whereas the indirect inhibition may occur through the stabilization of contact sites between outer and inner mitochondrial membranes (resulting in a reduced permeabilization of the outer mitochondrial membrane) and reduced cytochrome c and ROS release [162,164]. Antioxidative effects may also be achieved by stimulating glycolytic ATP production and limiting ATP consumption by mitochondria [165]. In addition to its effect on MPTP opening, HKII affects other mitochondrial parameters such as mitophagy, which is stimulated upon the reduction of mitochondrial HKII, as shown by the recruitment of Parkin in neonatal rat cardiomyocytes [155]. Moreover, HKII modestly increases oxygen consumption [166]. In contrast, the chronic reduction in mitochondrial HKII in heterozygous HKII-deficient mice is without effect on respiration [166]. In the context of cardioprotection, most studies focus on the role of HKII in IPC. Compared to rat hearts perfused under normoxic conditions, IPC enhances the mitochondrial HKII activity [157]. When measuring both HKI and II activity and using hearts undergoing I/R as controls, IPC is without effect on the mitochondrial HK activity in one study [167], but is increased in another study [168]. The aforementioned data also show that the mitochondrial protein amount of HKII is augmented at reperfusion in hearts undergoing IPC and that this effect is accompanied by a decrease in cytosolic HKII [168]. IPC prevents the I/R-induced loss of mitochondrial HKII in rat hearts in vitro and, accordingly, the induction of HKII dissociation from the mitochondria blocks the cardioprotection by IPC [157]. Despite similar reductions in mitochondrial HKII, the use of the TAT-HKII peptide prevents the protective effect of IPC, whereas IPC efficiently reduces myocardial damage in heterozygous HKII-deficient mice [169]. The reason for these discrepancies are unclear, but it is speculated that the TAT-HKII peptide interferes with mitochondrial binding sites involved in mitochondrial protein import. Whereas the TAT-HKII peptide is suggested to have effects on the vasculature independently from the mitochondrial dissociation of HKII [157], others question the hypothesis that TAT-HKII administration evokes vasoconstriction resulting in ischemia [170]. In the context of IPostC, measurement of the cytosolic HK activity indicated no difference between postconditioned and non-postconditioned isolated rat hearts, which was in line with the similar HK amounts in total protein extracts [171]. Pharmacological pre- and postconditioning with the cAMP analog 8-Br-cAMP-AM, however, protected against I/R damage by binding HKII to mitochondria and inhibiting MPTP opening [172].

In patients undergoing elective first-time on-pump isolated coronary artery bypass graft surgery, a RIC protocol is without protective effects on the release of cardiac troponin T and C-reactive protein. In the corresponding atrial tissue samples, mitochondrial HKII levels and protein activities are similar between control and RIC-treated patients. Moreover, no changes in the phosphorylation of AMPK and AKT are induced by RIC; however, it has to be stated that the study is too underpowered to prove its primary goal: the reduction in cardiac troponin T release by RIC [173].

In summary, myocardial I/R injury induces a dissociation of HKII from the mitochondria and IPC prevents such loss. The preserved amounts of mitochondrial HKII contribute to the reduction in myocardial damage via the prevention of apoptosis, the reduction in ROS formation, and the inhibition of MPTP opening at reperfusion [164]. The mitochondrial localization and activity of HKII are, at least in part, regulated by other kinases such as AKT and GSK3β, which also partially reside in the organelles. Data on the role of HKII in other cardioprotective maneuvers such as IPostC or RIC are sparse. One (presumably underpowered) clinical study failed to demonstrate an involvement of mitochondrial HKII in RIC. Therefore, further studies and an alternative to the TAT-HKII peptide in order to modify HKII localization are needed to clarify the contribution of mitochondrial HKII in the protection from myocardial I/R injury.

### 4.3. Adenosine Monophosphate-Activated Protein Kinase (AMPK)

AMPK exists as a heterotrimeric holoenzyme formed by the catalytic subunit α, the scaffolding subunit β, and the regulatory subunit γ and is expressed in essentially all eukaryotic cells [174]. The protein functions as a cellular energy sensor and restores energy homeostasis in response to increased ATP consumption or decreased ATP production [174,175]. Within cardiomyocytes, AMPK activity is involved in a variety of cellular processes including glucose and lipid metabolism, protein synthesis, apoptosis, and autophagy [176]. During ischemia, AMPK becomes activated via the binding of AMP or phosphorylation at threonine 172 and stimulates glucose utilization and glycolytic ATP production [177]. Hypoxic H9C2 cells also show increased AMPK phosphorylation [178]. Additionally, activation of AMPK occurs in response to excessive amounts of ROS (for a review, see [179,180]), and the pharmacological activation of AMPK is associated with decreased infarct size after myocardial I/R injury [181,182]. However, data also show a lack of AMPK activation upon I/R [183]. The already stimulated activation of AMPK with ischemia is further enhanced by IPC [184], RIC [185], and hypoxic postconditioning [186]. The preconditioning cycles of I/R are sufficient to induce AMPK activity [187]. Compared to sole I/R, AMPK phosphorylation increases upon acute but not delayed RIC [188] and also upon IPostC [189]. In contrast to the aforementioned data, a lack of increased AMPK phosphorylation by IPC [190] and the absence of effects of the pharmacological inhibition of AMPK by compound C on the cardioprotection by IPostC [191] are also described.

Mitochondrial function is regulated by AMPK in several aspects including mitochondrial biogenesis, fission and fusion, the removal of damaged mitochondria by mitophagy [178,192], and MPTP opening [181]. All of these mitochondrial functional parameters are modified by cardioprotective maneuvers. The regulation of mitochondrial function by AMPK suggests that a least a certain fraction of the protein is localized within the organelles and will directly modify mitochondrial function. Indeed, AMPK is identified in mitochondria isolated from mouse embryonic fibroblasts [193,194], L6 myotubes [195], kidney, liver, gastrocnemius muscle, and heart [53]. A detailed analysis on subfractionated mitochondria indicates that the protein localizes to the outer mitochondrial membrane [53]. Phosphorylated AMPK is enriched in mitochondria isolated from muscle cells and targets AKAP, whereby a direct interaction between AMPK and AKAP has not been demonstrated yet. However, the AMPK-induced phosphorylation of AKAP facilitates mitochondrial respiration [195]. The mitochondrial fission factor MFF, which is an outer membrane receptor for the fission protein Drp1, also represents a mitochondrial protein phosphorylated by AMPK [35]. The MFF phosphorylation induced upon AMPK phosphorylation may prepare the cells to initiate mitophagy [35]. The importance of AMPK for mitochondrial fission is confirmed in HEK293 cells, where the protein ARMC10 (Armadillo repeat-containing protein 10) is phosphorylated at serine 45 by AMPK. The knockout of ARMC10 prevents mitochondrial fission stimulated by AMPK activation [196].

**Figure 3:**
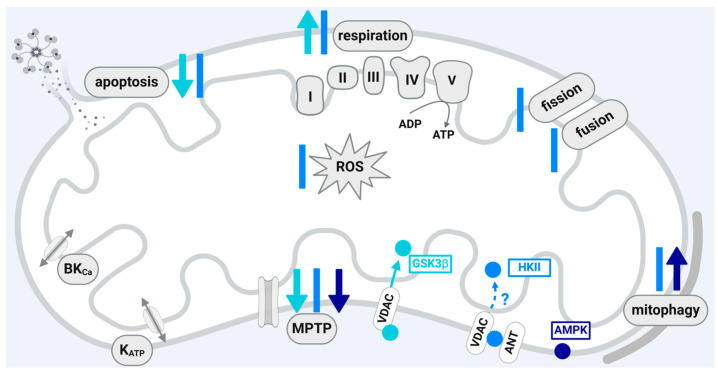
Effects of mitochondrially localized protein kinases associated with the RISK pathway on the mitochondrial function of cardiomyocytes or cardiomyocyte cell lines. The figure summarizes the effects of mitochondrially localized glycogen synthase kinase 3β (GSK3β, turquoise), hexokinase II (HKII, light blue), and adenosine monophosphate-activated protein kinase (AMPK, dark blue) on mitochondrial function. Arrows pointing upwards indicate an activating effect upon a cardioprotective stimulus; arrows pointing downwards indicate an inhibiting effect upon a cardioprotective stimulus; lines without an arrowhead indicate influence on mitochondrial function without cardioprotective stimulus; light blue dotted arrow with question mark refers to an alternative localization of HKII within the mitochondrial matrix; for further details, see Table 2. Created with BioRender.com. Abbreviations: I, II, II, IV, V (ATP synthase) indicate respiratory chain complexes; ADP: adenosine diphosphate; ANT: adenine nucleotide transporter; ATP: adenosine triphosphate; BK_Ca_: Ca^2+^-activated potassium channel; K_ATP_: ATP-dependent potassium channel; MPTP: mitochondrial permeability transition pore; ROS: reactive oxygen species; VDAC: voltage-dependent anion channel.

Taken together, the available data point to a mitochondrial localization of AMPK in several organs or cell types and to a contribution of the protein towards cardioprotection (see Figure 3). However, it is unclear whether the cardioprotective strategies include a translocation of the protein to the mitochondria and whether the signaling cascades include and are dependent on the translocation of AMPK to the mitochondria. Without such proof of a causal significance of mitochondrial AMPK for cardioprotection, a conclusive evaluation of the mitochondrial fraction of protein in this context is not possible.

A scheme that summarizes the influences of GSK3β, HKII, and AMPK localized within the mitochondria of cardiomyocytes or cardiomyocyte cell lines on the function of the organelles is shown in Figure 3.

## 5. Protein Kinases Not Assigned to the RISK or NO/PKG Pathways

In the following section, we describe two proteins, C-Jun N-terminal kinase and p38 MAPK, that are clearly involved in myocardial I/R injury, but are not assigned to one of the classical protective signaling pathways such as the RISK or NO/PKG pathway. As Src seems to be downstream of JNK, we discuss the kinase in the following section. With PTEN-induced putative kinase 1 (PINK1), we discuss a protein whose main function relates to mitophagy.

### 5.1. C-Jun N-Terminal Kinase (JNK)

JNK is a member of the mitogen-activated protein kinase (MAPK) family [197]. It becomes transiently phosphorylated and activated upon I/R and contributes to myocardial damage [198]. Accordingly, the use of JNK inhibitors reduces myocardial I/R damage [198,199,200]; however, an aggravation of myocardial I/R damage is also observed upon inhibition of the JNK pathway [201]. Whether the activation of JNK mediates protective or deleterious effects seems to be dependent on the duration of ischemia and the bioenergetic state of the postischemic myocardium [202]. The complexity of the consequences of JNK phosphorylation or dephosphorylation is emphasized in the context of cardioprotection, where ischemic and pharmacological preconditioning increase, whereas ischemic or pharmacological postconditioning decrease JNK phosphorylation [198,203,204]. JNK phosphorylation, which is enhanced by myocardial I/R, is reduced by RIC [205]. The detrimental effects of JNK activation during myocardial I/R are mediated, e.g., by the induction of mitochondrial dysfunction, including the activation of apoptosis [198,206] and ROS generation [200]. The influence of JNK on mitochondrial function suggests that the protein may exert its function directly within the organelles. Indeed, JNK is detected in mitochondria isolated from different cells or organs, including human umbilical vein endothelial cells [207], HeLa cells [208], lung [207], liver [209,210], brain [211,212], and heart [206,213]. Within mitochondria, JNK is present in the outer mitochondrial membrane, where it binds and phosphorylates the mitochondrial membrane scaffold protein SAB (SH3 domain-binding protein that preferentially associates with Bruton’s tyrosine kinase) [54]. The docking of JNK to SAB induces an intramitochondrial signal transduction cascade leading to impaired respiration and, thus, increased ROS formation [214,215,216]. ROS then activate apoptosis signal-regulating kinase, which targets mitogen-activated protein kinase 4 and 7, thereby creating a P-JNK/SAB/ROS activation loop with continuous JNK activation, finally inducing cell death [216]. The P-JNK/SAB/ROS activation loop is initiated upon different stressors including drug toxicity, a high-fat diet, immune attack, or endoplasmic reticulum stress [207,216]. Within cardiac myocytes, oxidative stress causes JNK activation, which, in turn, leads to the release of cytochrome c from the mitochondria, thereby inducing apoptosis [206]. In rat hearts in vivo, infarct size is reduced after the inhibition of JNK activity by SR-3306. Here, the protective effect of JNK inhibition is mediated via mitochondrial JNK, as the inhibition of the protein–protein interaction between JNK and SAB reduces oxidative stress and, finally, infarct size after 30 min ischemia and 24 h reperfusion [200]. Increased JNK phosphorylation is also observed upon the activation of mammalian STE20-like kinase 1, leading to the mitochondrial translocation of the fission protein dynamin-related protein (Drp)1, which, in turn, causes excessive mitochondrial fission, ROS formation, and apoptosis [217]. It is suggested that I/R injury decreases the protein amounts of dual-specificity protein phosphatase 1, which activates JNK. The subsequent increase in the JNK-mediated transcription of the mitochondrial fission factor (MFF) finally leads to excessive mitochondrial fission, apoptosis, and cell death [218]. However, the complex role of JNK in myocardial I/R injury is demonstrated in rat hearts in vitro, where the cardioprotection by IPC is associated with increased mitochondrial amounts of JNK [213].

JNK exerts a complex role in myocardial I/R injury including protective or deleterious effects depending on the experimental conditions. Within mitochondria, JNK is present in the outer mitochondrial membrane. The inhibition of mitochondrial JNK reduces infarct size due to the reduction in oxidative stress, whereas the stimulation of mitochondrial JNK enhances excessive fission and apoptosis, which finally induces cell death. The conditions favoring the mitochondrial import of JNK, the exact protein–protein interactions of the protein within the organelles, and the corresponding function of mitochondrial JNK in the context of I/R injury need to be addressed in further studies.

### 5.2. Src-Family Protein Tyrosine Kinases (SFKs)

In cardiomyocytes, seven members of the SFKs, a subfamily of non-receptor tyrosine kinases, are expressed: Fyn, Fgr, Yes, Src, Lyn, Lck, and Blk. Among them, the activation of Src and Lck, which appears to be distal to PKCε, is associated with IPC in rabbit hearts [219] and in isolated rabbit cardiomyocytes [220]. The finding that the combined inhibition of PKC and protein tyrosine kinase interferes with the cardioprotection by IPC in pigs [89] suggests that downstream signaling pathways, including proteins such as Src, may also be suppressed.

Although SFKs are permanent residents of cytoplasm, Src, Fyn, Lyn, and Fgr are also localized in the mitochondria of several cell types [221], including the heart [59]. In human and bovine cell lines, Src translocation to mitochondria seems to be dependent on anchoring proteins (AKAP)121 [57] or docking protein 4 [60]. In H9C2 cells, simulated hypoxia/reoxygenation decreases mitochondrial Src phosphorylation, an effect prevented by the inhibition of JNK [20]. Also, the blocking of SAB reverses the hypoxia/reoxygenation-induced dephosphorylation of mitochondrial Src [222]. In rodent hearts, cardioprotection by IPC causally involves mitochondrial Src tyrosine 416 phosphorylation upon reperfusion, with Src and phospho-Src located in complex I of the electron transport chain and phospho-Src associated with a reduction in complex I activity and ROS formation [55]. Of note, in contrast to the other cardioprotective kinase signaling pathways, Src appears to reduce complex I activity. However, the effects of Src on respiration appear to be cell-type-dependent, since Src also increases the activity of complexes of the electron transport chain in rat brain mitochondria [223]. Src-dependent tyrosine phosphorylation of the adenine nucleotide translocator 1 (ANT1)—a member of the mitochondrial carrier family relevant for mitochondrial metabolism—seems to be linked to cardioprotection via isoflurane-induced preconditioning [58]. Morphin-induced cardioprotection increases mitochondrial Src phosphorylation at reperfusion [224]. Cardioprotection by exogenous NO at reperfusion reduces oxidative stress through the Src-mediated inhibition of complex I at reperfusion [225].

In summary, Src interacts with complex I of the electron chain in rodent heart mitochondria, suggesting a localization of the protein at the inner mitochondrial membrane. Data demonstrating interactions of mitochondrial Src in HEK293 cells with matrix proteins indicate that the function of mitochondrial Src is not restricted to the inner mitochondrial membrane [56]. Considering that SFKs affect mitochondrial function in cell types other than cardiomyocytes through different pathways [221] and that the co-localization of SFKs with mitochondria is also evident in human cells, it is reasonable to assume that additional, yet unknown, SFK-dependent signaling cascades involving mitochondria may be relevant for cardioprotection. While a role of Src in the cardioprotection by late IPC is known [226], it is still unclear whether a mitochondrial fraction of Src is involved in this process. The effects of Src within mitochondria are inadequately investigated and further studies are needed to elucidate the role of the mitochondrial fraction of the protein in myocardial I/R injury.

### 5.3. p38 Mitogen-Activated Protein Kinases (p38 MAPK)

p38 MAPK is not included in the classical cardioprotective intracellular signaling pathways [2,13,70]; nevertheless, p38 MAPK is activated through and causally involved in cardioprotection by IPC in rodents [227], rabbits [228], and pigs [229], and possibly also in RIC, where a pharmacological p38 inhibition abrogates the protection in rats [230]. The two p38 MAPK isoforms α and β seem to have different or even opposing functions [231]. Pharmacological p38 MAPK inhibition and knockout mouse experiments identify that p38 MAPK α activation during the preconditioning stimulus is causal to mediate IPC [232] whereby p38 MAPK α activation during myocardial ischemia aggravates injury [233]. The increased activity of p38 MAPK β during sustained ischemia is associated with reduced infarct size in pigs undergoing IPC [229]. In isolated rat neonatal cardiomyocytes, the activation of p38 MAPK α during ischemia triggers apoptosis, whereas p38 MAPK β is responsible for pro-survival signaling during preconditioning [234]. In chick embryonic ventricular cells, p38 MAPK localizes to mitochondria and p38 MAPK inhibition blocks ceramide-induced apoptosis [235]. Specifically, p38 MAPK β resides in mitochondria isolated from rat neonatal cardiomyocytes and interacts with the MnSOD [62]. The interaction between p38 MAPK β and MnSOD is confirmed in mitochondria from adult female mice, where p38 MAPK β phosphorylates MnSOD at threonine 79 and serine 106 [61]. However, since mitochondria have not been subfractionated in this study, it is not clearly shown whether p38 is localized in the mitochondrial matrix as is MnSOD. The activation of MnSOD by p38 MAPK β decreases ROS formation and is implicated in the cardioprotection of 17β-estradiol [61]. The mitochondrial localization of p38 MAPK is not restricted to the β isoform, and p38α is also detected in mitochondria isolated from rat hearts [236]. The activation of p38 MAPK during I/R is compartmentalized: whereas during ischemia, p38 MAPK is activated through the protein kinase C (PKC) isoform ε within mitochondria, p38 MAPK activity is increased in cytosolic, mitochondrial, and membrane fractions during reperfusion [43,236]. A non-isoform-specific p38 MAPK inhibitor, given before or during ischemia in rodent hearts, attenuates mitochondrial swelling, mitochondrial ROS generation, and mitochondrial membrane potential depolarization, whereas it fails to prevent the loss of mitochondrial function when given at the onset of reperfusion [237]. The concurrent inhibition of p38 MAPK α and p38 MAPK β in all of these reports, however, complicates the interpretation of the exact role of p38 MAPK, so the specific function of mitochondrial p38 MAPK remains elusive. The pharmacological inhibition just indicates that p38 MAPK is crucial for mitochondrial function during I/R [238]. Given the lack of evidence for a role of p38 MAPK in the cardioprotection of the human heart and the known opposing function of the p38 MAPK isoforms, it is not surprising that a clinical trial with an oral, non-isoform-specific p38 MAPK inhibitor in patients with acute non-ST-elevation myocardial infarction was neutral in terms of reducing infarct size as measured by troponin I release [239].

In sum, whereas data point to a mitochondrial localization of p38 MAPK α and β in myocardial cells, the exact submitochondrial localization of the proteins has not been established so far. It is hypothesized that mitochondrial p38 MAPK contributes to cardioprotection via reduced ROS formation; however, the impact of mitochondrial p38 MAPK on the function of the organelles under physiological conditions and in the context of I/R injury needs to addressed in further and more detailed studies.

### 5.4. PTEN-Induced Putative Kinase 1 (PINK1)

As described above, AMPK plays a role in the cardioprotective signaling pathways by stimulating mitophagy and, thereby, the removal of damaged mitochondria from their cellular pool. Mitophagy proceeds in both PINK1/Parkin-dependent and -independent pathways. PINK1 is a serine/threonine kinase, which, under physiological conditions, is imported into the mitochondria, cleaved by the protease presenilin-associated rhomboid-like protein, and then translocated into the cytosol and degraded by the proteasome, resulting in low protein amounts of the kinase [240]. The PINK1/Parkin-dependent pathway is stimulated in dysfunctional mitochondria with a reduced membrane potential, which leads to the inhibition of PINK1 hydrolysis. PINK1 translocates to the outer mitochondrial membrane of mitochondria with reduced membrane potential via the TOM complex, undergoes dimerization and autophosphorylation, and then becomes activated [63,64,65,241]. Upon PINK1 activation, the E3 ubiquitin ligase Parkin is recruited from the cytosol to the mitochondria via PINK1-induced downstream phosphorylation events [64,66]. Parkin, in turn, polyubiquitylates proteins of the outer mitochondrial membrane such as VDAC (voltage-dependent anion channel) and Mfn1 and Mfn2 involved in the fusion of mitochondria. The subsequent degradation of mitofusin stimulates mitochondrial fission and induces mitophagy [242]. PINK1/Parkin-independent pathways to induce mitophagy are reviewed in detail elsewhere [240,243,244].

The effects of PINK1 on mitochondrial function are not restricted to mitophagy; rather, they also include other functional parameters important for the outcome of myocardial I/R injury and the protection from it. The analysis of mitochondrial function in isolated cardiomyocytes from PINK1-deficient mice demonstrates reduced mitochondrial respiration and membrane potential and susceptibility to MPTP opening, whereas ROS formation is stimulated in cells undergoing simulated I/R [245].

Infarct size increases after I/R in PINK1-deficient mouse hearts, showing that the absence of PINK1 enhances the vulnerability of the heart towards a damaging insult [245]. Consequently, the overexpression of PINK1 in HL1 cells reduces cell death induced by simulated I/R [245]. The permanent ligation of the left anterior descending coronary artery (LAD) in Parkin-deficient mice leads to increased mortality, and the surviving animals are characterized by impaired heart function and decreased mitophagy [246]. Mitophagy is initiated by myocardial I/R injury in rat hearts in vivo, as indicated by the increased expression of PINK1 and Parkin [247,248,249,250]. The amounts of PINK1 are also induced in the mitochondria of H9C2 cells subjected to hypoxia/reoxygenation [250] and upon permanent LAD ligation in mice [251]. Here, the small GTPase RhoA plays a role in the stabilization of mitochondrial PINK1 by interacting with PINK1 at the mitochondria [251,252]. PINK1 expression and its function in mitophagy are also controlled by microRNA-421 [253]. In contrast to the aforementioned studies showing the increased expression of PINK1 in models of myocardial I/R injury, reduced amounts of PINK1 are described in neonatal rat cardiomyocytes [254] and in the mitochondria of H9C2 cells subjected to hypoxia/reoxygenation [255].

Even though data demonstrate that the initiation of autophagy protects the heart against I/R injury [256] and the activation of PINK1 by IPC in kidneys [257] or by RIC in the rat brain [258], studies on the role of PINK1 in cardioprotection by IPC, RIC, or IPostC are still lacking. However, some studies address the role of cardiac PINK1 in pharmacological preconditioning and show increased PINK1 expression in cardiomyocytes upon the administration of remifentanil [254], whereas the cardioprotection by activating aldehyde dehydrogenase 2 is associated with reduced amounts of PINK1 [250]. Pharmacological postconditioning with triiodothyronine further elevates the already increased PINK1 amounts in neonatal rat cardiomyocytes undergoing simulated I/R [259]. Additionally, acetylcholine given to H9C2 cells at reoxygenation enhances the mitochondrial amounts of PINK1 and stimulates mitophagy [255].

While it is generally assumed that the activation of mitophagy is beneficial in myocardial I/R injury and is activated by cardioprotective maneuvers, excessive mitophagy—which seems to depend on the duration of I/R—may also have detrimental effects [15]. PINK1 is important for mitophagy, but also for other mitochondrial functions affected by myocardial injury and the protection from it. However, direct evidence that PINK1 is activated by IPC, RIC, or IPostC in the heart is still lacking. Further studies should aim to investigate PINK1 as a putative target protein of cardioprotective strategies.

**Figure 4:**
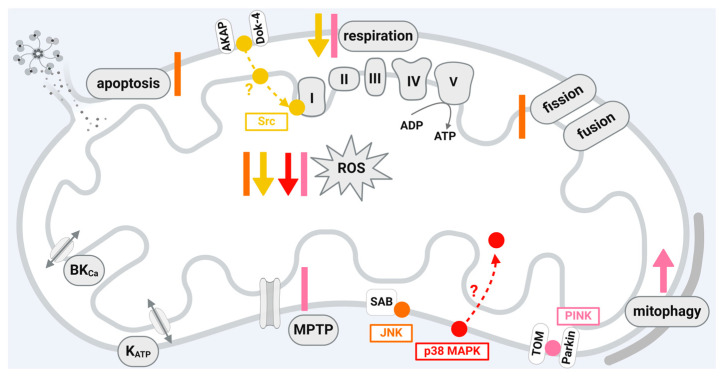
Effects of mitochondrially localized protein kinases not associated with the RISK or NO/PKG pathway on mitochondrial function of cardiomyocytes or cardiomyocyte cell lines. The figure summarizes the effects of mitochondrially localized glycogen synthase kinase C-Jun N-terminal kinase (JNK, orange), Src (yellow), p38 mitogen-activated protein kinase (p38 MAPK, red), and PTEN-induced putative kinase 1 (PINK1, pink) on the mitochondrial function of cardiomyocytes or cardiomyocyte cell lines. Arrows pointing upwards indicate an activating effect upon a cardioprotective stimulus; arrows pointing downwards indicate an inhibiting effect upon a cardioprotective stimulus; lines without an arrowhead indicate influence on mitochondrial function without cardioprotective stimulus; yellow and red dotted arrows with question marks refer to alternative localizations of p38 MAPK and Src within the matrix; for further details, see text. Created with BioRender.com. Abbreviations: I, II, II, IV, V (ATP synthase) indicate respiratory chain complexes; ADP: adenosine diphosphate; AKAP: PKA-anchoring protein; ATP: adenosine triphosphate; BK_Ca_: Ca^2+^-activated potassium channel; Dok-4: downstream of kinase 4; K_ATP_: ATP-dependent potassium channel; MPTP: mitochondrial permeability transition pore; ROS: reactive oxygen species; SAB: SH3 domain-binding protein that preferentially associates with Bruton’s tyrosine kinase; TOM: translocase of the outer membrane.

A scheme that shows the influences of JNK, Src, p38 MAPK, and PINK1 localized in cardiomyocytes or cardiomyocyte cell lines on mitochondrial function is given in Figure 4.

## 6. Conclusions

Cardioprotective strategies such as IPC, RIC, and IPostC activate signaling transduction pathways, which finally lead to the reduction in myocardial damage upon I/R injury. The beneficial effects of cardioprotection are partly achieved by the maintenance of normal mitochondrial function in terms of respiration, ROS formation, MPTP opening, mitochondrial dynamics, ion homeostasis, apoptosis, and mitophagy. The signal transduction pathways stimulated by IPC, RIC, and IPostC include protein kinases, of which a certain amount resides within mitochondria. An overview of the localization, translocation, and protein–protein interactions of these mitochondrially localized protein kinases is given in Table 1. The mitochondrial translocation and activities of these kinases are modified by myocardial I/R injury and regulate mitochondrial function. The effects of these mitochondrially localized kinases on mitochondrial function in the context of cardioprotection are summarized in Figure 2, Figure 3 and Figure 4 and Table 2.

**Table 2:** Mitochondrially localized protein kinases in cardiomyocytes or cardiomyocyte cell lines and their functional effects.

Protein Kinase	Experimental Model		Functional Effects
	Preparation	Stimulus	
PKA	adult guinea pig cardiomyocytes without vs. with pharmacological PKA activation	-	prevention of mitochondrial Ca^2+^ overload [77]
	mitochondria, mitoplasts from cattle heart	-	activation of mitochondrial complex I respiration [37]
	permeabilized adult rat cardiomyocytes	-	increased mitochondrial ROS generation [78]
	H9c2 rat cardiomyocytes with in vitro H/R, rat myocardium and mitochondria from WT mice myocardium vs. myocardium of mice expressing activated PKCε with in vitro I/R	without vs. with NO	Drp1-dependent reduction of mitochondrial fission [39]
	adult and neonatal rat cardiomyocytes with in vitro H/R	without vs. with HC	GSK3β-dependent inhibition of MPTP opening [74]
PKCε	mitochondria from myocardium of WT mice vs. of mice with transgene expression of activated PKCε	-	PKCε/VDAC-dependent reduction of apoptosis [43] and inhibition of MPTP opening [45]
	neonatal rat cardiomyocytes with in vitro H/R and without and with pharmacological PKCε specific translocation inhibitor	without vs. with HC	cytochrome c oxidase dependent PKCε translocation, improved mitochondrial respiration [44]
	mitochondria from rabbit hearts with in vitro I/R	without vs. with IC	increased K_ATP_ opening [97]
	mitochondria, mitoplasts from rat hearts without vs. with pharmacological PKCε activation	-	increased K_ATP_ opening and reduced ROS formation [98]
PKCδ	mitochondria from rat hearts with in vitro I/R, without vs. with pharmacological inhibition of PKCδ translocation	-	decreased mitochondrial ROS formation and improved mitochondrial respiration [108]
	rat hearts with in vitro I/R, without vs. with pharmacological inhibition of PKCδ translocation	-	reduction of apoptosis via release of cytochrome c [107]
GSK3β	mitochondria from WT mice and mice with permanently activated GSK3β with in vivo I/R, without and with pharmacological GSK3β inhibition	without vs. with IC	increased respiration, inhibition of MPTP opening [128]
	adult and neonatal rat cardiomyocytes with in vitro H/R	without vs. with HC	inhibition of MPTP opening and apoptosis, enhanced mitochondrial biogenesis [74]
	adult cardiomyocytes and mitochondria from mouse hearts without vs. with pharmacological GSK3β inhibition and neonatal rat cardiomyocytes	-	increased mitochondrial respiration and ATP production [130]
HKII	neonatal rat cardiomyocytes with Ca^2+^ and H_2_O_2_-treatment without vs. with pharmacological AKT activation and mouse mitochondria with Ca^2+^-treatment without vs. with recombinant kinase active AKT	-	inhibition of MPTP opening—decreased release of cytochrome c [50]
	adult rat cardiomyocytes without vs. with pharmacological enhancement of mitochondrial HKII binding with recombinant GSK3β	-	inhibition of MPTP opening [126]
	HeLa cells and adult rat cardiomyocytes without vs. with peptide displacing HKII from mitochondria	-	enhanced MPTP susceptibility to ROS [152]
	mitochondria from rat hearts without vs. with the cAMP analogue 8-Br-cAMP-AM	-	inhibition of MPTP opening [172]
	mitochondria from neonatal rat cardiomyocytes with in vitro H and mitochondria from mouse hearts with in vivo I without vs. with AAV9-mediated expression of mitochondrial HKII dissociating peptide	-	increased Parkin-mediated mitophagy [155]
	mouse hearts in vitro perfused without vs. with HKII peptide reducing mitochondrial HKII	-	increased mitochondrial respiration [166]
AMPK	H9c2 rat cardiomyocytes with in vitro H	without vs. with pharmacological AMPK activation	induced mitophagy [178]
	adult rat cardiomyocytes with mechanical stress	without vs. with pharmacological AMPK activation	inhibition of mPTP opening [181]
JNK	mitochondria from H9c2 rat cardiomyocytes and primary human cardiomyocytes without vs. with in vitro H_2_O_2_/FeSO_4_-treatment, neonatal rat cardiomyocytes without vs. with in vitro H_2_O_2_/FeSO_4_-treatment, mitochondria from rat hearts without vs. with in vivo I/R	-	increased ROS formation [200]
	mitochondria from rat hearts without vs. with in vitro JNK-activation	-	increased cytochrome c release [206]
	adult cardiomyocytes from hearts of WT mice and Mst KO mice without vs. with in vitro H and without vs. with in vivo I	-	enhanced fission [217]
	hearts of WT mice vs. DUSP1 KO mice with in vivo I/R	-	enhanced fission [218]
SFKs (Src)	mitochondria from adult rat cardiomyocytes with in vivo I/R	without vs. with IC	decreased mitochondrial respiration during IC, reduced ROS generation [55]
	adult rat cardiomyocytes with in vitro H/R	without vs. with NO	decreased complex I activity, reduced ROS generation [225]
p38 MAPK	mitochondria from hearts of WT and Ovx mice/ER null mice with in vivo I/R	without vs. with 17β-estradiol	p38 MAPKβ decreased ROS formation [61]
	mitochondria from rat hearts with in vivo I/R	pharmacological p38 MAPK inhibition	attenuated mitochondrial swelling, mitochondrial ROS generation, and mitochondrial membrane potential depolarization [237]
PINK1	HL-1 mouse cardiomyocytes, WT vs. with PINK1 over-expression with mechanical stress and adult cardiomyocytes from WT vs. PINK1-deficient mice	-	inhibition of MPTP opening, decreased mitochondrial membrane potential, reduced mitochondrial respiration, increased ROS [245]
	adult and H9c2 rat cardiomyocytes with in vitro H/R mitochondria from WT vs. PINK1-deficient mice	without vs. with acetylcholine at reoxygenation	increased mitophagy [255]

Abbreviations: AAV9: adeno-associated virus 9; AKT: protein kinase B; AMPK: adenosine monophosphate-activated protein kinase; ANT: adenine nucleotide transporter; BAG5: B cell lymphoma (BCL)2-associated athanogene 5; BNIP: BCL2/adenovirus E1B 19 kDa protein-interacting protein 3; Drp1: dynamin-related protein 1; DUSP1: dual specificity protein phosphatase 1; ER: endoplasmatic reticulum; ERK: extracellular signal-regulated kinase; GSK3β: glycogen synthase kinase 3 β; H: hypoxia; HC: hypoxic conditioning; HKII: hexokinase II; H/R: hypoxia/reoxygenation; I: ischemia; IC: ischemic conditioning; I/R: ischemia/reperfusion; JNK: C-Jun N-terminal kinase; K_ATP_: ATP-dependent potassium channel; MPTP: mitochondrial permeability transition pore; Mst KO: 3-mercaptopyruvate sulfurtransferase; NO: nitric oxide; Ovx: ovariectomy; p38 MAPK: p38 mitogen-activated protein kinases; PINK1: PTEN-induced putative kinase 1; PKA: protein kinase A; PKC: protein kinase C; ROS: reactive oxygen species; SAB: SH3 domain-binding protein that preferentially associates with Bruton’s tyrosine kinase; SFK: Src-family protein tyrosine kinases; VDAC: voltage-dependent anion channel; WT: wild type.

Whereas, in general, the positive effects of cardioprotective strategies on mitochondrial function are relatively well described, the contribution of protein kinases present within mitochondria is less clear, and systematic analyses to elucidate the influence of mitochondrial protein kinases on the function of the organelles in I/R injury and the protection from it are lacking. These ambiguities concern possible differences regarding the localization of kinases in mitochondrial subpopulations such as subsarcolemmal or interfibrillar mitochondria. In addition, whether the function of mitochondrial protein kinases is specific for certain species is currently unknown. Furthermore, it is unclear whether and how the mitochondrial part of the kinases is involved in the known cardioprotective signaling pathways. With regard to the translation of preclinical data to the clinical situation, it is necessary to define the exact site of action of the involved protein kinases. Only the characterization of the precise functions of the kinases involved in cardioprotection, including their subcellular sites of action, will allow the proteins to be used as therapeutic targets in order to reduce myocardial I/R damage.

## Figures and Tables

**Table 1:** Localization, translocation, and protein–protein interactions of mitochondrial protein kinases.

Protein Kinase	Mitochondrial Localization	Translocation through	Interaction with
PKA	OMM: murine embryonic fibroblasts [35], mouse brain mitochondria [36]; IMM and matrix: bovine heart mitochondria [37]; rat heart mitochondria [38]	n.d.	AKAP: mouse brain mitochondria [36] ETC complex I: bovine heart mitochondria [37] Drp1: rat hearts [39]
PKCε	IMM: rat heart mitochondria [40] IMM and matrix: rat heart mitochondria [41]	HSP90-TOM20: rat heart mitochondria [40] HSP90-TOM70: rat cardiomyocytes [42]	JNK, p38 MAPK, ERK: mouse heart mitochondria [43] Cyt c oxidase subunit 4: neonatal rat cardiomyocytes [44] VDAC, ANT, HKII: mouse heart mitochondria [45]
GSK3β	possibly OMM: rat heart mitochondria [46]	VDAC2: H9C2 cells [47]	ANT: rat heart mitochondria [48] VDAC2: rat heart mitochondria [46]
HKII	OMM: reviewed in [49]	n.d.	AKT: neonatal rat cardiomyocytes [50]; transfected neonatal rat cardiomyocytes [51]; VDAC: HL1 cells [52], reviewed in [49]
AMPK	OMM: mouse gastrocnemius muscle mitochondria [53]	n.d.	MFF: transfected human embryonic kidney–293 T cells [35]
JNK	possibly OMM: mouse hepatocytes [54]	n.d.	SAB: mouse hepatocytes [54]
SFKs	IMM: rat heart mitochondria [55] Possibly matrix: HEK293 cells [56]	n.d.	AKAP121: GC2 cells [57] ANT1: rat heart mitochondria, HeLa cell mitochondria [58] ETC complex I: rat heart mitochondria [55] ETC complexes I and III: rat heart mitochondria [59] Dok-4: transfected bovine aortic endothelial cells [60] Diverse matrix proteins: HEK293 cells [56]
p38 MAPK	possibly matrix: mouse heart mitochondria [61]	n.d.	MnSOD: neonatal rat cardiomyocytes [62], mouse heart mitochondria [61] PKCε: mouse heart mitochondria [43]
PINK1	OMM: HeLa cell mitos [63], reviewed in [64]	TOM complex: HeLa cell mitochondria [63], reviewed in [65]	E3 ubiquitin ligase Parkin complex: transduced Flp-In T-Rex HEK293 cells [66], reviewed in [64]

Abbreviations: AKAP: PKA-anchoring protein; AKT: protein kinase B; AMPK: adenosine monophosphate-activated protein kinase; ANT: adenine nucleotide transporter; Dok-4: downstream of kinase 4; Cyt c: cytochrome c; Drp1: dynamin-related protein 1; ERK: extracellular signal-regulated kinase; ETC: electron transport chain; GSK3β: glycogen synthase kinase 3 β; HKII: hexokinase II; HSP90: heat shock protein 90; IMM: inner mitochondrial membrane; JNK: C-Jun N-terminal kinase; MFF: mitochondrial fission factor; MnSOD: manganese superoxide dismutase; n.d.: not determined; OMM: outer mitochondrial membrane; p38 MAPK: p38 mitogen-activated protein kinases; PINK1: PTEN-induced putative kinase 1; PKA: protein kinase A; PKCε: protein kinase C ε; SAB: SH3 domain-binding protein that preferentially associates with Bruton’s tyrosine kinase; SFK: Src-family protein tyrosine kinases; TOM: translocase of the outer membrane; VDAC: voltage-dependent anion channel.
